# The impact of environmental policy on soil quality: Organic carbon and phosphorus levels in croplands and grasslands of the European Natura 2000 network

**DOI:** 10.1016/j.jenvman.2018.06.003

**Published:** 2018-10-01

**Authors:** Andrea Hagyó, Gergely Tóth

**Affiliations:** a European Commission, Joint Research Centre, Sustainable Resources Directorate, Via Enrico Fermi 2749, I - 21027 Ispra VA, Italy; b University of Pannonia, Georgikon Faculty, Department of Soil Science and Environmental Informatics, H-8360, Keszthely, Deák Ferenc u. 16, Hungary; c Hungarian Academy of Sciences, Centre for Agricultural Research, Institute for Soil Science and Agricultural Chemistry, H-1022, Budapest, Herman O.u.15, Hungary

**Keywords:** Natura 2000, Europe, Soil organic carbon, Phosphorus, LUCAS dataset, Low intensity farming

## Abstract

In this study, the Natura 2000 nature protection network of the European Union is assessed in the context of soil quality management. We explore the hypothesis that the soil quality of croplands and grasslands — as indicated by soil organic carbon and phosphorus levels — is better in terms of environmental parameters within the Natura 2000 network than outside it.

The soil organic carbon and phosphorus content of 479 cropland and 450 grassland sites within Natura 2000 were compared with their nearest neighbours outside the network. The comparisons were repeated for pairs of both cropland and grassland sites by soil texture groups.

The study revealed that organic carbon content was significantly higher in Natura 2000 sites than in non-protected areas for both croplands and grasslands. For croplands, this was true only for those with signs of ploughing, whereas for croplands without signs of ploughing there were no significant differences between Natura 2000 and non-protected areas.

Areas with sand and loamy sand soils had significantly higher soil organic carbon content within the Natura 2000 network than outside it, for both croplands and grasslands. This was the only texture class that showed a significant difference in the case of croplands, whereas three further texture groups had higher soil organic carbon content in Natura 2000 grassland sites than on grassland sites outside this network.

There was no significant difference in soil phosphorus content between areas within the Natura 2000 network and non-protected areas, except for grasslands with light textured soils, where soil phosphorus levels were significantly lower within Natura 2000 sites than outside them.

The results suggest that the management of croplands and grasslands of Natura 2000 sites tends to perform better than that of adjacent areas with similar land cover in terms of soil carbon conservation. The difference is more evident for sites with certain soil characteristics. On the other hand, the nutrient input - as determined by phosphorus levels - of Natura 2000 croplands and grasslands generally does not appear to be less intensive than that of surrounding areas outside the network.

## Highlights

• Natura 2000 programme contributes to soil organic carbon conservation.
• Majority of Natura 2000 croplands&grasslands has >5% more organic carbon than outside.
• Soil phosphorus levels do not differ in and outside Natura 2000 at EU level.
• Nutrient input of Natura 2000 areas in general is not less intensive than outside.

## 1 Introduction

The Natura 2000 nature protection network is the most important establishment of the European Union's (EU's) biodiversity strategy (Sundseth and Creed, 2008). Its aim is to protect Europe's most valuable and threatened species and habitats. As the below-ground environment hosts a large portion of terrestrial biodiversity (Jeffery et al., 2010), the role of Natura 2000 sites in preserving soil life should be recognised.

The EU's biodiversity protection strategy aims to preserve not only biodiversity but also ecosystem services. The agricultural areas of Natura 2000 sites can contribute to various ecosystem services, including global climate regulation, by preventing carbon loss from soils through sustainable management practices (European Commission, 2013).

About two thirds of terrestrial carbon is below ground (Batjes, 1996). In arable lands and in grasslands especially, most carbon accumulates in the soil and this carbon pool is much more stable than above-ground carbon pools. The role of SOC in reducing greenhouse gas emissions is therefore crucial. The Kyoto Protocol allows carbon emissions to be offset by the demonstrable removal of carbon from the atmosphere, including through the improved management of agricultural soils. Natura 2000 sites could therefore contribute to achieving the EU's objective of reducing greenhouse gas emissions by 80%, as compared with levels in 1990, by 2050.

Organic carbon benefits a range of soil-related ecosystem services and biodiversity (Jeffery et al., 2010). In this regard, Natura 2000 sites can play a role in improving biodiversity and assisting with climate regulation. Jantke et al. (2016) made the same suggestion, as they found significant overlap between Natura 2000 sites and regions with high soil carbon content across Europe.

In developed countries, including Member States of the European Union, phosphorus accumulated in agricultural soils due to the use of high doses of phosphorus fertilisers in the past decades (Lemercier et al., 2008). While the use of phosphorus-based fertilisers has improved crop yields in Europe, the negative side effects of increased levels of phosphorus in the ecosystem, such as eutrophication, have become a problem (Csathó and Radimszky, 2011). In light of the functions and multiple effects of soil phosphorus, it is especially important to manage its level in order to effectively maintain or improve the condition of ecosystems. Measures related to managing phosphorus levels in the Natura 2000 network are linked with the Water Framework Directive (WFD, 2000/60/EC).

SOC and phosphorus levels are two important indicators of soil quality (Bastida et al., 2008). Soil quality, in turn, has a distinct role in nutrient cycling, depending on the biophysical conditions of the site and soil management factors (Schröder et al., 2016). While soil quality is determined by many other internal and external factors, and its assessment depends on the purpose of the evaluation, phosphorus content and SOC levels are among the most widely assessed soil properties for soil quality evaluation and monitoring (Herrick et al., 2016). Phosphorus concentration in topsoil is one of the most relevant indicators of the extent of fertiliser application in croplands (Tóth et al., 2013b). Furthermore, below-ground biodiversity, an important constituent of soil quality and supplier of genetic diversity in general, is directly conditioned by the amount of decomposed and transformed organic matter in soil (Jeffery et al., 2010; Pimentel and Burgess, 2014; Orgiazzi et al., 2016).

Following the considerations described above, we analysed SOC and phosphorus levels to assess the condition of the soil within Natura 2000 sites at EU level. We assume that the low-intensity farming practices promoted in the Natura 2000 network lead to lower phosphorus levels in the soil and that no or minimum tillage leads to an increase in SOC content (Havlin et al., 1990; West and Post, 2002; Freibauer et al., 2004; Soussana et al., 2004; Quincke et al., 2007). The hypothesis that soils contain more organic carbon and less phosphorus in Natura 2000 sites than in non-protected areas was tested for both grasslands and croplands. SOC content and dynamics depend, to a great extent, on soil texture (Burke et al., 1989; Dexter, 2004; Ottoy et al., 2017), which was also taken into account in the study.

In agricultural areas in general, high phosphorus concentrations pose higher environmental risks than low phosphorus levels (Schoumans et al., 2015); therefore, in our study, lower phosphorus values were considered to indicate better soil condition.

As agricultural areas play an important role in reducing greenhouse gas emissions through increased carbon sequestration (Fornar et al., 2016; Minasny et al., 2017), we further analysed SOC contents by testing two possible mitigation objectives.

Although the optimisation of phosphorus levels in soil is a key factor for sustaining multiple ecosystem services, maintaining crop production capacity and preventing or reducing the pressure of nutrient enrichment on ecosystem condition, establishing optimisation scenarios was outside the scope of the current study.

Our study can be considered the first EU-wide evaluation of the performance of Natura 2000 specifically with regard to soil conservation and soil quality management in cropland and grassland sites.

## 2 Materials and methods

### 2.1 Materials

#### 2.1.1 Natura 2000 management

The management of agricultural areas in Natura 2000 is based on the principle of sustainable development and use (Sundseth and Creed, 2008). Maintaining the characteristic biodiversity of nearly half of the terrestrial part of the Natura 2000 network depends on the maintenance of low-intensity farming, for example by grazing semi-natural pastures and by mowing hay meadows (European Commission, 2014). An important part of the Natura 2000 network therefore relies on the activities of farmers and shepherds. All Natura 2000 network sites have a common goal, but the management practices are not uniformly prescribed and varies between sites. It was not within the scope of this study to evaluate site-specific management practices, but to assess the performance of the network at EU level.

#### 2.1.2 Soil data

We used the SOC and phosphorus data of soil samples from the Land Use/Cover Area Frame Survey (LUCAS) topsoil survey (Tóth et al., 2013a,b). The LUCAS programme (Martino and Fritz, 2008) is an EU-wide network of field surveys managed by the statistical office of the European Union (Eurostat) (http://ec.europa.eu/eurostat/web/lucas/overview). The aim of LUCAS is to collect harmonised data on land use and land cover, focusing on parameters relevant for agricultural policy.

Soil sampling was undertaken in 2009. The sampling design was based on the intersection of a regular 2 km × 2 km grid. Approximately 265,000 geo-referenced sampling points have been classified according to seven land cover classes. Land cover has been defined through the use of orthophotographs or satellite images. For the purpose of the soil module of the LUCAS 2009 survey, a representative sub-sample of around 200,000 points was selected from across the EU. At approximately 10% of these points, topsoil samples (taken at depths of 0–30cm) were taken and analysed in a central laboratory to create a coherent database (Tóth et al., 2013a,b). SOC content (g kg^−1^) was measured after dry combustion according to ISO 10694:1995. Phosphorus content (mg kg^−1^) was measured by spectrometric determination of phosphorus soluble in sodium hydrogen carbonate solution, following the ISO 11263:1994 method. A series of quality control procedures were applied during the development of the database (Tóth et al., 2013b).

**Fig. 1:**
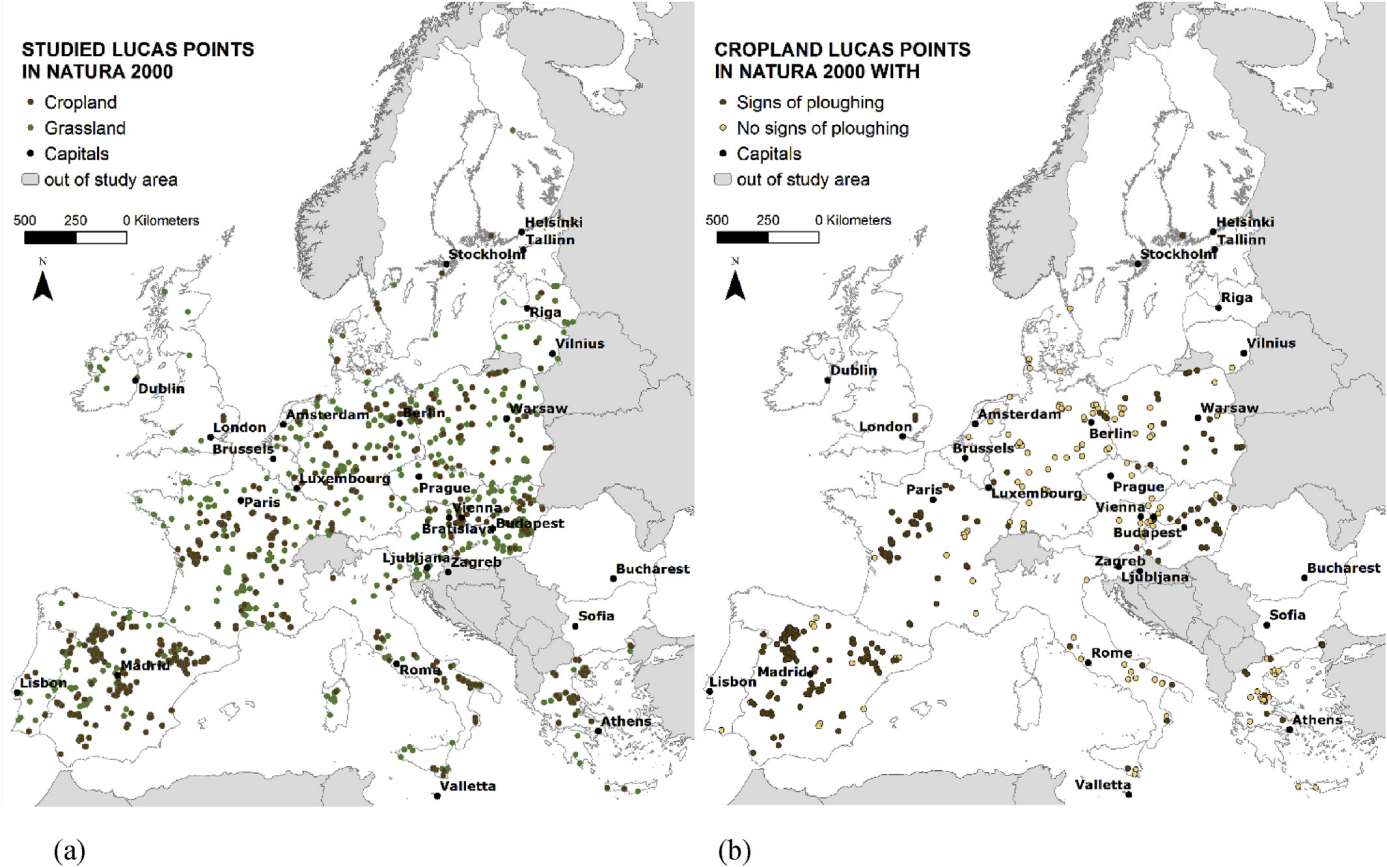
(a) Distribution of the LUCAS points studied within the Natura 2000 network. (b) Classification of cropland LUCAS points in Natura 2000 based on the binary management variable (signs of ploughing/no signs of ploughing).

For our study, we selected those LUCAS points that fell inside Natura 2000 sites and were covered by croplands or grasslands according to the land cover attribute of the LUCAS database (Fig. 1a). Each point was matched with the closest LUCAS point outside the Natura 2000 network that shared similar land cover properties (and, in the next step, similar soil properties as well). Only mineral soils were considered. All analyses were conducted using these pairs of points covering 23 Member States of the EU. Croatia, Romania, Bulgaria, Cyprus and Malta were excluded because of missing data.

### 2.2 Methods

SOC and phosphorus concentrations in croplands and grasslands inside and outside Natura 2000 areas were compared. The comparison was performed on the matched samples of measurement points inside and outside the Natura 2000 network, described above. All statistical analyses were performed using the R statistical software package (R Core Team, 2013).

SOC and phosphorus data were not normally distributed as defined by the Shapiro-Wilk normality test. The non-parametric Wilcoxon matched-pairs signed-rank test was therefore applied to assess whether or not the SOC and phosphorus contents of the matched samples differed. A 95% confidence level was applied to test the validity of the hypothesis.

Both cropland and grassland points were then classified by soil texture. Pairs of nearest neighbours inside and outside Natura 2000 sites with the same land cover and soil texture class were selected. The comparison was repeated for these pairs as well. The soil texture classes were defined based on the United States Department of Agriculture (USDA) soil texture classification system (USDA, 1975). Sand and loamy sand classes were grouped as one class, as were clay and silty clay classes. Silt and sandy clay textured soils were excluded from the study because of the low sample size.

With this approach, similarities in the main conditions (climate, land cover and physical soil properties) of phosphorus and SOC dynamics of the sampling sites compared were ensured. As both members of each pair of points came from the same region, the level of applied agro-technology, determined by the availability of machinery and material and labour inputs, and the degree of advancement of management techniques were considered similar.

In addition, we also examined the potential impact of the management-related variable available in the LUCAS database. This is a binary variable: the surveyor registered if any sign of ploughing could be seen in the cropland plot (by recording a ‘yes’ or ‘no’ response) (Fig. 1b). We divided the Natura 2000 cropland sites into two groups: (1) those with signs of ploughing (N = 235) and (2) those without signs of ploughing (N = 128). Pairs were matched based on this variable and the comparison was repeated for both groups.

The SOC contents of the Natura 2000 sites were further analysed to investigate the role of agricultural areas in reducing greenhouse gas emissions through increased SOC content. Two possible scenarios were tested, representing two possible greenhouse gas mitigation objectives of EU climate policy. The proportion of the sites studied in the Natura 2000 network that fulfilled each of two possible thresholds was determined. The two thresholds were a difference of more than 5 g kg^−1^ SOC and a difference of more than 5% in carbon content. The differences were calculated as the difference between the Natura 2000 site and the nearest neighbour outside the Natura 2000 area.

## 3 Results

### 3.1 Soil organic carbon and phosphorus content in croplands and grasslands

The median SOC content was found to be significantly higher within Natura 2000 sites than outside these areas (Table 1). The median phosphorus content was lower within Natura 2000 sites than outside these areas, but the differences were not significant.

**Table 1:** Results of the comparison of the soil organic carbon (SOC) and phosphorus (P) content in mineral topsoil of the study sites; *significant difference at 95% confidence level.

	SOC (g kg^−1^)					P (mg kg^−1^)				
	N	Median_in_	Median_out_	V-value	p-value	N	Median_in_	Median_out_	V-value	p-value
All croplands	479	13.3*	12.4	67416.5	0.0010	386	28.2	29.05	35768	0.5841
All grasslands	450	26.5*	21.1	65926.5	3.743e-08	324	24.55	26.9	23246	0.0825

### 3.2 Soil organic carbon and phosphorus content in croplands grouped by ploughing and by soil texture

Results of the separate analyses for cropland sites with and without ploughing showed that SOC content was significantly higher in Natura 2000 sites on only those fields that had been ploughed (Table 2). There was no significant difference between the SOC content of crop fields with no signs of ploughing within and outside Natura 2000 areas.

**Table 2:** Results of the comparison of the soil organic carbon (SOC) and phosphorus (P) content of croplands inside and outside Natura 2000 areas (Wilcoxon signed-rank test); *significant difference at 95% confidence level.

	SOC (g kg^−1^)					P (mg kg^−1^)				
	N	Median_in_	Median_out_	V-value	p-value	N	Median_in_	Median_out_	V-value	p-value
Croplands, clay & silty clay	42	17.95	16.4	437	0.861	31	26.2	24.3	294	0.2096
Croplands, clay loam	54	11.45	12.3	731	0.9245	42	19.25	23.8	321	0.1045
Croplands, silty clay loam	80	18.25	17.7	1352.5	0.2673	66	28.25	29.4	1201.5	0.5418
Croplands, sandy clay loam	19	6.4	9.7	57	0.1336	16	22.3	35.2	44	0.2312
Croplands, loam	77	14.55	12.85	1865.5	0.0650	57	25.1	30.9	664.5	0.1994
Croplands, silt loam	79	15.2	15.1	1785	0.3176	64	37.6	32.3	1175	0.2543
Croplands, sandy loam	82	8.95	8.55	1649	0.8913	71	30.5	40.3	953.5	0.0633
Croplands, sand & loamy sand	44	10.05*	7.8	695	0.0199	39	40.1	39.3	381	0.9066
Croplands with ploughing	235	11.5*	10.3	16,241.5	0.0228	192	26.95	27.6	9772	0.4302
Croplands without ploughing	128	16.35	15.0	4688	0.1833	107	36.5	40.0	2560.5	0.308

Climate is one of the major factors influencing SOC dynamics in soil; therefore, differences in climatic conditions could be drivers for different rates of divergence in SOC content. However, the mixed geographical distribution of sites with higher and lower levels of SOC did not support this hypothesis.

When furthering our analysis to investigate the possible effect of soil physical properties, we found that significant differences in SOC content could be observed only for soils with sand or loamy sand textures (Table 2; map in supplementary material).

Based on the studied pairs of LUCAS points, we found no significant difference in topsoil phosphorus content between mineral soils of croplands within and outside Natura 2000 areas (Table 2).

### 3.3 Soil organic carbon and phosphorus content in grasslands grouped by soil texture

In contrast to the observations made with regard to croplands, not only light but also medium and heavier textured soils tended to have higher SOC levels in grasslands within Natura 2000 areas than outside them. Grasslands with sand/loamy sand, loam, sandy clay loam and clay/silty clay soils showed significantly higher SOC contents within Natura 2000 areas than outside them. As with the case of croplands, climatic factors can be excluded because of the heterogeneity of the situations.

When investigating differences in topsoil phosphorus content between grasslands within and outside Natura 2000 areas, based on the studied pairs of LUCAS points, we found significant differences only in the case of light textured soils (Table 3), which had significantly lower phosphorus contents in Natura 2000 sites than outside them.

**Table 3:** Results of the comparison of the soil organic carbon (SOC) and phosphorus (P) content of grasslands within and outside Natura 2000 areas (Wilcoxon signed-rank test); *significant difference at 95% confidence level.

	SOC (g kg ^−1^)					P (mg kg ^−1^)				
	N	Median _in_	Median _out_	V-value	p-value	N	Median _in_	Median _out_	V-value	p-value
Grasslands, clay & silty clay	46	45.25*	34.7	811	0.0032	35	30.5	29.7	354	0.5329
Grasslands, clay loam	30	34.15	33.95	230	0.9677	13	27.3	28.0	41	0.7869
Grasslands, silty clay loam	67	34.6	31.6	1108.5	0.8513	46	23.5	26.4	525	0.8698
Grasslands, sandy clay loam	10	32.85	15.15	43	0.1309	10	19.0	32.4	22	0.625
Grasslands, loam	63	27.1*	24.5	1311.5	0.0380	44	24.3	20.3	626	0.1287
Grasslands, silt loam	64	26.6*	22.65	1379	0.0236	50	19.4	23.7	591	0.657
Grasslands, sandy loam	101	20.1	16.5	2786	0.4768	71	28.6	28.1	1164	0.5155
Grasslands, sand & loamy sand	66	14.0*	11.6	1419	0.02375	55	28.3*	42.6	515	0.0330

### 3.4 Testing policy-based carbon sequestration scenarios

The organic carbon content was higher by more than 5 g kg^−1^ in 27% of the cropland sites within Natura 2000 areas than on the nearest neighbouring sites outside Natura 2000. A total of 53% of the Natura 2000 cropland sites fulfilled the objective of having a carbon content more than 5% higher than the corresponding neighbouring site (Table 4). The proportions for grasslands were 47% and 58%, respectively.

**Table 4:** Number of cases where the topsoil organic matter (SOC) content is (1) higher, (2) higher by more than 5 g kg^−1^ or by more than 5%, and (3) lower by more than 5 g kg^−1^ or by more than 5% within Natura 2000 sites than outside.

	N (sample size)	Mean_SOC_ in Natura 2000	STD_SOC_ in Natura 2000	(1) SOC (Natura 2000) > SOC (normal)	(2) SOC (Natura 2000) > SOC (normal) by > 5 g kg^−1^/>5%	(3) SOC (Natura 2000) < SOC (normal) by > 5 g kg^−1^/>5%
Croplands	479	15.85	9.80	272	130/252	94/186
Grasslands	450	32.65	23.08	279	212/263	125/166

STD: standard deviation.

On the other hand, there were pairs where SOC content was lower within the Natura 2000 site than in the corresponding non-protected site of similar land cover. Nevertheless, the evaluation of the differences, either an increase or a decrease, has to be considered in relation to the local biophysical conditions.

**Fig. 2:**
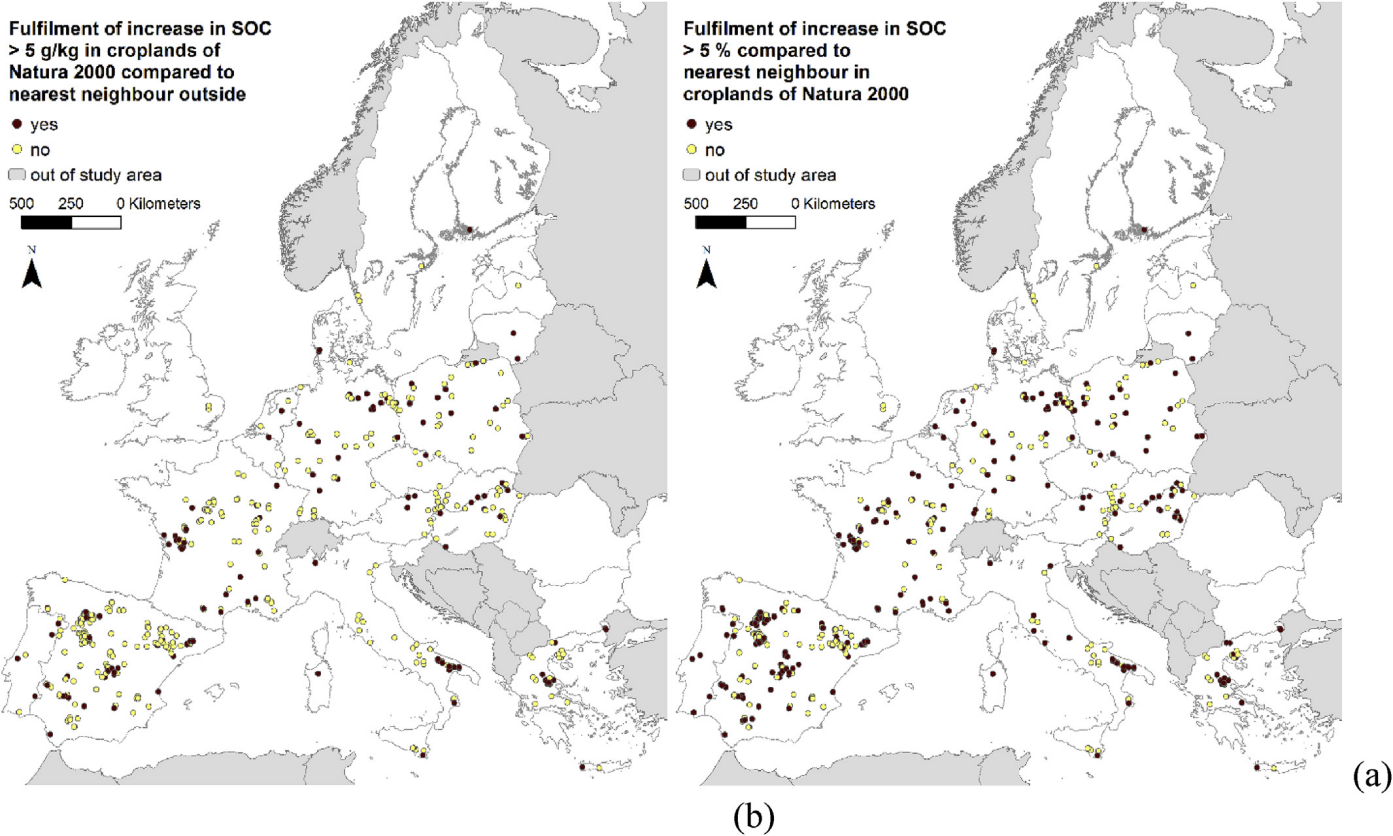
Spatial distribution of cropland LUCAS points within Natura 2000 areas that fulfil the increment thresholds of (a) 5 g kg^−1^ and (b) 5% in organic carbon content compared with the nearest neighbour cropland points.

The sampling sites that have higher level of SOC by more than 5 g kg^−1^ or 5% are not concentrated in any particular regions, but rather are distributed evenly throughout the study area (Fig. 2).

## 4 Discussion

The Natura 2000 network was created using an approach that considers humans an integral part of nature and recognises that the two work best in partnership with one another (European Commission, 2014). Most of the agricultural areas in Natura 2000 are marginal and have been managed by extensive farming systems that have, in most cases, developed over time, with farm structures and farming practices closely adapted to local conditions. These serve as examples of good practice in the management of farmlands in the Natura 2000 network.

While SOC and phosphorus concentrations are indicators of soil quality from both productivity and environmental perspectives, their values can have different meanings depending on pedoclimatic conditions and different soil functions. For instance, high phosphorus levels can enhance productivity but can also present a risk for load to surface- and sub-surface water systems, depending on biophysical conditions and soil management practices. While we acknowledge the complexity of the relationships of SOC and phosphorus with soil functions and ecosystems in general, the assessment of carbon and phosphorus cycles and their agronomic and environmental consequences was outside the scope of the current study.

A general comparison between areas of traditional agriculture and extensively managed low-fertility high-nature-value farmlands (Gardi et al., 2016) showed higher levels of organic carbon in areas with low-intensity cultivation. This finding is in line with our results based on the specific comparisons between Natura 2000 sites and the corresponding sites outside the network in the same region.

Our results suggest that, in general, the management practices applied in Natura 2000 sites in both croplands and grasslands increase SOC content.

However, when considering soil texture types, it is also apparent that significant differences can be observed only in sand and loamy sand textured soils in croplands. Sampling points with these kinds of textures are concentrated mainly in the postglacial areas south of the Baltic coast and in western Iberia. One possible explanation for this relationship can be that climatic conditions in the southern Baltic and to some degree in the western Iberian area allow a higher ratio of accumulation to decomposition of organic carbon (Freibauer et al., 2004), however, it was not supported by assessing the data by geographical location at different climatic zones. This suggests that, apart from climatic and management factors, soil properties can play a role in differentiated humus sequestration, which leads to higher rates of organic carbon accumulation in these soils. Although, in similar environments, clayey soils generally bind organic carbon in higher amounts than sandy soils (Stevenson, 1994), the relative loss of carbon following land use change is lower in clayey soils (Burke et al., 1989). Recuperation of SOC can therefore be faster in lighter textured soils, following the introduction of soil management practices that enhance SOC levels.

In the grassland areas of Natura 2000, not only sand and loamy sand but even heavier textured soils have higher SOC contents than grasslands in non-protected areas. One possible explanation for this could be the higher amounts of residues of primary biomass production in Natura 2000 sites. As management recommendations for Natura 2000 require extensive grazing regimes with low to moderate stocking levels (European Commission, 2014), the proportion of ungrazed biomass in the Natura 2000 network is most probably higher than on areas outside the network. Because the biomass productivity potential of soils with medium to heavier textures is higher, because of their favourable water retention characteristics, the biomass left on land is likely to be higher too. Higher biomass production with increased root biomass and above-ground residues will lead to a higher degree of organic carbon accumulation in soil. Nevertheless, as a result of lack of data on biomass and a small sample size of soils with different textures, we could not investigate this hypothesis further.

The soil management of croplands has also been found to have an impact on SOC content. The results suggest that higher SOC content is related to the soil management practices used on those fields, where techniques other than no-till techniques are applied.

Our analysis also extended to soil phosphorus content. As changes in phosphorus levels can occur relatively quickly, i.e. over only a few years when altering nutrient management on agricultural land, the similarity between phosphorus levels within and outside the Natura 2000 sites suggests that phosphorus fertiliser inputs are similar. This result might indicate that, in general, the management of Natura 2000 sites concentrates on organic-matter-conserving soil tillage and/or residue management and crop rotation practices, whereas phosphorus fertilisers are used at similar levels to those used outside Natura 2000 sites. Nevertheless, an interesting connection between lower phosphorus levels and higher organic carbon levels on sandy and loamy sand soils was observed in grasslands. This might confirm that less-intensive agricultural practices will increase organic carbon within Natura 2000 grassland sites and, in parallel, decrease phosphorus levels in soil.

In addition, the present study has shown that the tested potential policy objectives of achieving a 5 g kg^−1^ or 5% increase in SOC content have been fulfilled on a remarkable proportion of the studied Natura 2000 sites compared with their nearest neighbour sampling points. However, soil properties can have an influence on this.

It would be very interesting to compare the biomass production of Natura 2000 sites and non-Natura 2000 areas to see whether or not there is a difference in crop output between sites with similar nutrient management but different soil and residue management practices. In accordance with the findings of Körschens et al. (2013), who compared long-term field experiments in Europe, it is very likely that switching cultivation methods to fit Natura 2000 requirements would not result in yield losses. As similar yields can be coupled with organic carbon sequestration, Natura 2000 sites can be regarded as facilitating the successful transition from conventional to sustainable agriculture, both from the viewpoints of biodiversity conservation and climate regulation.

## 5 Conclusions

The results obtained based on the LUCAS EU-wide soil survey suggest that the Natura 2000 programme enhances SOC content in croplands and grasslands. However, this does not apply in all cases and depends on local soil conditions. With regard to nutrient management, the croplands and grasslands of Natura 2000 do not differ from those outside the network at EU level, except in certain conditions.

The increase in SOC levels can help facilitate the goal of the Natura 2000 programme to protect and enhance biodiversity, at least as far as below-ground biodiversity is concerned. In addition to this benefit, the alternative soil management practices within Natura 2000 sites can also increase the sequestration of atmospheric carbon.

Continued efforts are needed to further improve the environmental parameters of the soil quality of Natura 2000 croplands and grasslands.

The results of the current study can be considered as baseline information on the performance of Natura 2000 sites with regard to soil quality. Based on the repeated LUCAS soil surveys, possibly including soil biodiversity parameters, this assessment can be repeated in the future in order to monitor progress towards fulfilling the aims of the Natura 2000 network.
