# Supplementary figures and images for: The impact of environmental policy on soil quality: Organic carbon and phosphorus levels in croplands and grasslands of the European Natura 2000 network
Source: J Environ Manage. 2018 Oct 1;223:9–15. doi: 10.1016/j.jenvman.2018.06.003 (PMC6087710; doi:10.1016/j.jenvman.2018.06.003)

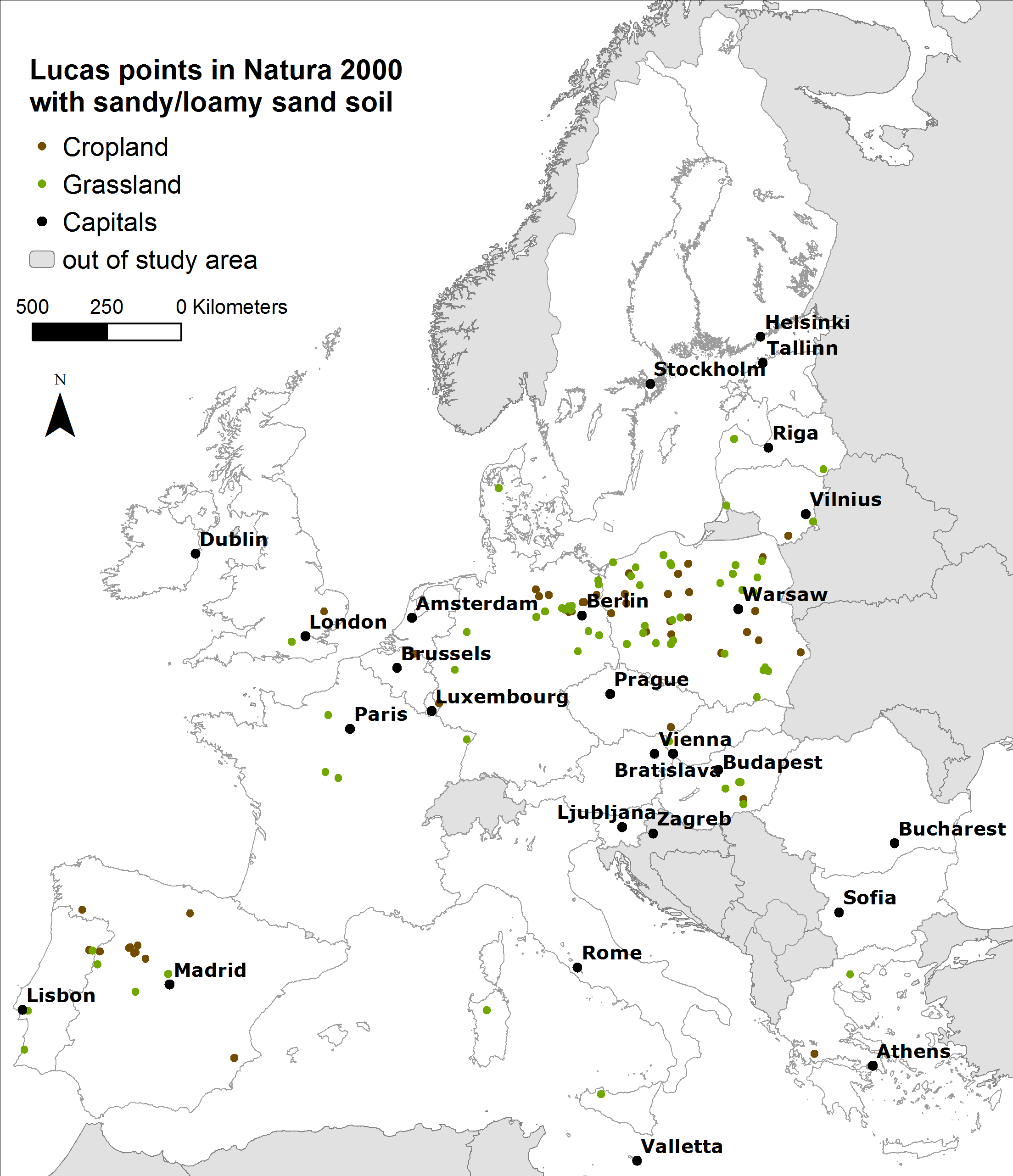


The studied LUCAS sampling points with sand/loamy sand soil.

Supplement: Multimedia component 1 [file mmc1.docx]
